# Interneuron odyssey: molecular mechanisms of tangential migration

**DOI:** 10.3389/fncir.2023.1256455

**Published:** 2023-09-14

**Authors:** Ikram Toudji, Asmaa Toumi, Émile Chamberland, Elsa Rossignol

**Affiliations:** ^1^Centre Hospitalier Universitaire (CHU) Sainte-Justine Research Center, Montréal, QC, Canada; ^2^Department of Neurosciences, Université de Montréal, Montréal, QC, Canada; ^3^Department of Biochemistry and Molecular Medicine, Université de Montréal, Montréal, QC, Canada; ^4^Department of Pediatrics, Université de Montréal, Montréal, QC, Canada

**Keywords:** GABA, interneurons, migration, blood vessels, oligodendrocytes, cytoskeleton, neurodevelopmental disorders, therapy

## Abstract

Cortical GABAergic interneurons are critical components of neural networks. They provide local and long-range inhibition and help coordinate network activities involved in various brain functions, including signal processing, learning, memory and adaptative responses. Disruption of cortical GABAergic interneuron migration thus induces profound deficits in neural network organization and function, and results in a variety of neurodevelopmental and neuropsychiatric disorders including epilepsy, intellectual disability, autism spectrum disorders and schizophrenia. It is thus of paramount importance to elucidate the specific mechanisms that govern the migration of interneurons to clarify some of the underlying disease mechanisms. GABAergic interneurons destined to populate the cortex arise from multipotent ventral progenitor cells located in the ganglionic eminences and pre-optic area. Post-mitotic interneurons exit their place of origin in the ventral forebrain and migrate dorsally using defined migratory streams to reach the cortical plate, which they enter through radial migration before dispersing to settle in their final laminar allocation. While migrating, cortical interneurons constantly change their morphology through the dynamic remodeling of actomyosin and microtubule cytoskeleton as they detect and integrate extracellular guidance cues generated by neuronal and non-neuronal sources distributed along their migratory routes. These processes ensure proper distribution of GABAergic interneurons across cortical areas and lamina, supporting the development of adequate network connectivity and brain function. This short review summarizes current knowledge on the cellular and molecular mechanisms controlling cortical GABAergic interneuron migration, with a focus on tangential migration, and addresses potential avenues for cell-based interneuron progenitor transplants in the treatment of neurodevelopmental disorders and epilepsy.

## 1. Introduction

Brain function requires the balanced and coordinated activity of excitatory glutamatergic projection neurons and cortical inhibitory GABAergic interneurons. The cortical excitatory projection neurons, often referred to as pyramidal cells, are generated from dorsal progenitors located in the pallium. On the other hand, cINs are generated from several progenitor pools located outside the pallium, in the ventral telencephalon (subpallium; Anderson et al., 1997; Wonders and Anderson, 2006). Consequently, pyramidal cells and cINs follow distinct migratory journeys during embryonic development to converge in the mature cerebral cortex. Pyramidal cells migrate radially over relatively short distances into the developing cortical plate, whereas cINs follow a complex process involving multiple consecutive phases: a tangential migration from their embryonic origin to the pallium, a switch to radial migration with intracortical dispersion and subsequent integration in their final laminar allocation in the cortex (Marin and Rubenstein, 2001; Marin, 2013). Mounting evidence suggests that alterations in cIN development or function contributes to the pathogenesis of several neurodevelopmental and psychiatric disorders including autism spectrum disorders (Vogt et al., 2018; Chen et al., 2020; Smith et al., 2020a; Amegandjin et al., 2021; Nomura, 2021; Juarez and Martinez Cerdeno, 2022), intellectual deficiency/learning disabilities/attention deficit disorders (Lupien-Meilleur et al., 2021; Ferguson et al., 2023), epilepsy (Rossignol et al., 2013; Jiang et al., 2016, 2018; Tran et al., 2020; Gertler et al., 2022; Ryner et al., 2023) and schizophrenia (Sohal and Rubenstein, 2019; Shen et al., 2021). Furthermore, *in utero* ethanol exposure was recently shown to disrupt cIN migration in a mouse model of fetal alcohol spectrum disorder (FASD; Skorput et al., 2015; Lee et al., 2022), suggesting that environmental factors, together with perturbations of intrinsic molecular programs, both play critical roles in cIN development, relevant to a range of neurodevelopmental disorders. This review aims to summarize some of the molecular and environmental mechanisms regulating cIN migration.

## 2. Extrinsic guidance cues directing cIN migration

### 2.1. Repulsion from the proliferative zone and onset of migration

The majority of cINs arise in the embryonic subpallium from multipotent progenitors in the medial (MGE) and caudal (CGE) ganglionic eminences, while a smaller fraction originates from the preoptic area (POA). MGE lineages produce parvalbumin- and somatostatin-expressing cINs which account for ~70% of the total GABAergic cIN population, while CGE-derived INs expressing the serotoninergic receptor 5-HT-3A comprise ~30% of the total cortical interneuron population (Fogarty et al., 2007; Lee et al., 2010; Miyoshi et al., 2010; Rubin et al., 2010; Yu et al., 2021). Regardless of their embryonic origin, newborn interneurons adopt a highly polarized morphology as they initiate their tangential migration, and they display an astonishing ability to move and interact with environmental cues: they extend, branch and remodel their leading process, retract unselected branches and orient in space in response to chemoattractant and repulsive cues. Although multiple mechanisms are shared between MGE and CGE-derived cINs, recent evidence suggests that distinct transcriptional programs regulate the migration and laminar positioning of CGE-derived cINs (Murthy et al., 2014; Miyoshi et al., 2015; Touzot et al., 2016; Wei et al., 2019; Limoni et al., 2021; Venkataramanappa et al., 2022). This review focuses on mechanisms governing MGE-derived cIN migration, which have been more extensively studied.

**Figure 1:**
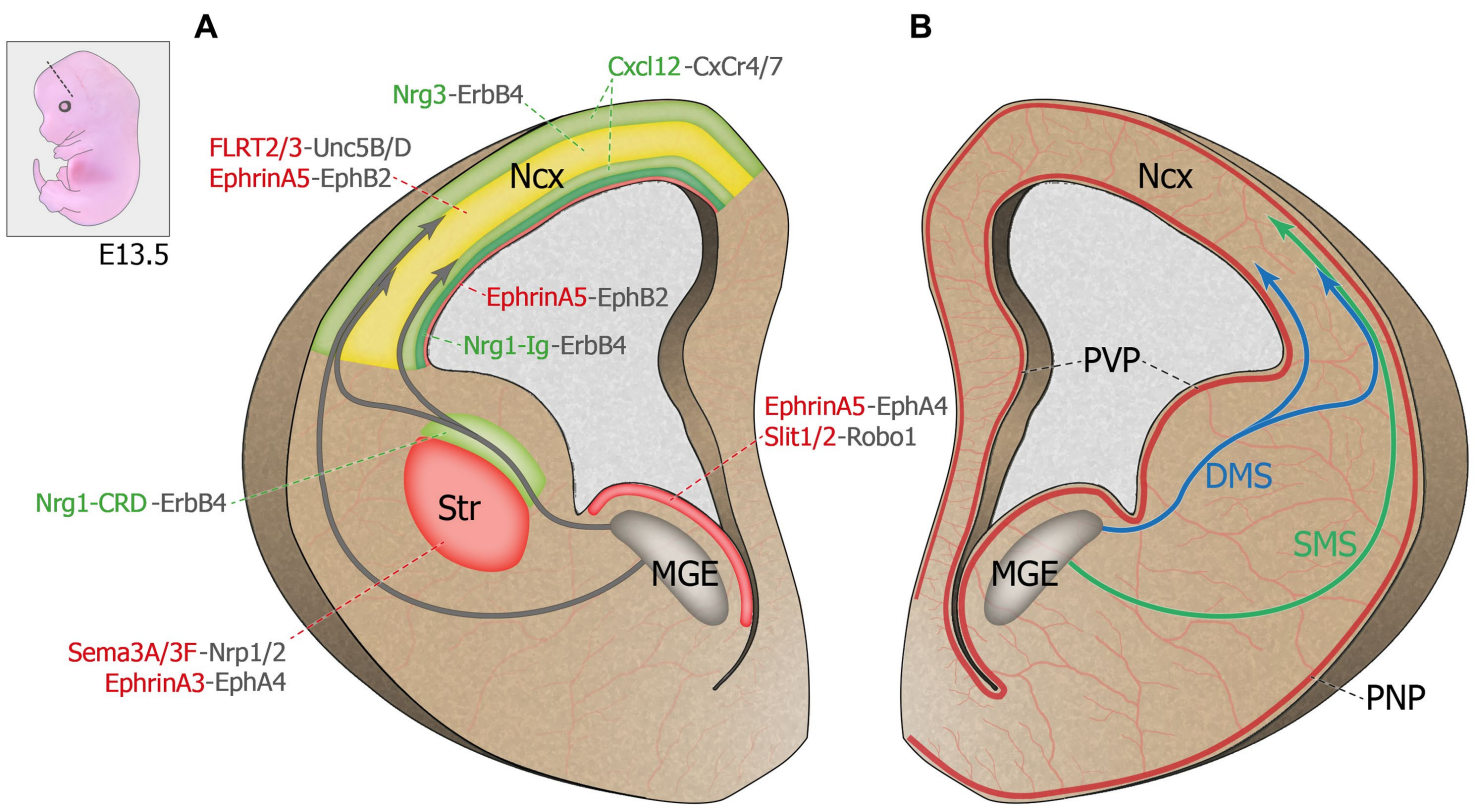
Molecular regulation of cortical interneuron migration. Top left corner: Representation of an E13.5 mouse embryo. Dash line illustrates the coronal plane through the telencephalon. **(A)** Schematic representation of a coronal hemi-section (left) highlighting the chemorepulsive (red) and chemoattractive (green) molecules guiding MGE-derived INs migrating towards the developing cortical plate. The cortical plate hosts both chemoattractive and chemorepulsive cues and is represented in yellow. **(B)** Schematic representation of a coronal hemi-section (right) showing the spatial proximity between the developing brain principal vascular networks and the MGE-derived IN migratory streams. DMS: deep migratory stream. E13.5: Embryonic day 13.5. MGE: medial ganglionic eminence. Ncx: neocortex. PNP: perineural vascular plexus. PVP: periventricular vascular plexus. SMS: superficial migratory stream. Str: striatum.

Newborn postmitotic cINs are actively repulsed from the proliferative zone by guidance cues expressed within the MGE ventricular (VZ) and subventricular (SVZ) zones, which triggers the onset of cIN tangential migration (Zhu et al., 1999; Marin et al., 2001; see Figure 1A). The diffusible cues Slit homologs 1 and 2 (Slit1 and 2), expressed in the VZ and SVZ zones of the MGE, were shown to repulse cINs *in vitro* and were though to contribute to the onset of migration away from the VZ and towards the cortical plate (CP; Yuan et al., 1999; Zhu et al., 1999). However, migration of cINs was unaffected in *Slit1^−/−^* and *Slit2^−/−^* mutant mice, although the repulsive effect of ventral structures remained (Marin et al., 2003), suggesting that other factors originating from the basal forebrain contribute to the initiation of cIN migration. Ephrins and their receptors Eph tyrosine kinases appear critical in this process. EphrinA5, expressed in the VZ, exerts a repulsive effect on migrating cINs expressing the EphA4 receptor, contributing to VZ avoidance (Zimmer et al., 2008). In addition to repulsive cues, cINs also encounter motogenic cues that stimulate their motility. For instance, EphrinA2 expressed by cINs interacts with its EphA4 receptor expressed by glial cells and exerts a reverse signaling effect that increases the speed of cIN migration (Steinecke et al., 2014). Other motogenic factors that promote cIN migration include hepatocyte growth factor/scatter factor (HGF/SF; Powell et al., 2001), brain-derived neurotrophic factor (BDNF) and neurotrophin 4 (NT4; Polleux et al., 2002), and glial-derived neurotrophic factor (GDNF; Pozas and Ibanez, 2005).

En route to the dorsal pallium, cINs must avoid entering the striatum to pursue their dorsal migration, contrary to striatal interneurons, which end their trajectory in this structure [see review (Villar-Cervino et al., 2015)]. The avoidance of the striatum by cINs is mostly due to the chemorepulsive effect of semaphorin 3A and 3F, expressed in the striatal mantle, as well as EphrinA3, expressed by striatal cells (Marin et al., 2001; Rudolph et al., 2010). Indeed, migrating interneurons destined to populate the cortex, but not those directed to the striatum, express the semaphorin receptors neuropilin 1 and 2 (Nrp1 and 2) and the EphA4 receptor. Thus, they are directed away from the striatum in response to semaphorin 3A/3F (Marin et al., 2001) and EphrinA3 (Rudolph et al., 2010). Interestingly, the loss of the Slit receptor Roundabout homolog 1 (Robo1) leads to a failure of this repulsive effect, resulting in an aberrant accumulation of cINs in the developing striatum (Andrews et al., 2006), a phenotype that was absent from *Slit1^−/−^* and *Slit2^−/−^* mutants (Marin et al., 2003), suggesting that Robo signaling regulates cIN migration independently of Slits. Indeed, it was since shown that the repulsive effect of semaphorin 3A/3F requires binding of Robo1 to Nrp1, such that the loss of Robo1 function in cINs leads to their aberrant accumulation in the striatum through a loss of sensitivity to the repulsive effect of semaphorins 3A/3F (Hernández-Miranda et al., 2011).

### 2.2. Guidance towards the dorsal pallium

Chemoattractive molecules create permissive corridors for migrating cINs (see Figure 1A). Neuregulin-1 (Nrg1), a protein containing an epidermal growth factor (EGF)-like motif which dimerizes and activates transmembrane tyrosine kinases related to the EGF receptor, was the first factor described as having a chemoattractive effect on migrating cINs (Flames et al., 2004). The *NRG1* gene, identified as a schizophrenia susceptibility gene [as reviewed in Rossignol, 2011; Marin, 2012], is subject to alternative splicing (Falls, 2003), resulting in the expression of two distinct protein isoforms in the developing telencephalon: Nrg1-Ig, a diffusible protein expressed in the pallium, and Nrg1-CRD, a membrane-bound protein expressed along the dorsal migratory streams, which, respectively, act as long- and short-range attractors for MGE-derived INs. The Nrg1 receptor tyrosine-protein kinase ErbB4, a member of the epidermal growth factor receptor family, is required for this process as interneurons lacking ErbB4 largely fail to enter the Nrg1-CRD+ corridor as they migrate towards the cortex (Flames et al., 2004). Interestingly, ErbB4 is not ubiquitously expressed in all migrating cINs, suggesting that different subtypes of cINs might be guided by distinct extracellular factors (Yau et al., 2003). Recent work has revealed that Nrg1/ErbB4-mediated chemoattraction of migrating cINs involves two molecular cascades: PI3-kinase/PTEN/AKT and p35/Cdk5, which both play keys roles in cIN migration (Rakić et al., 2015). Like Nrg1, ErbB4 receptor exists in two different isoforms, one with a binding site for PI3-kinase (cyt1) and one without (cyt2). At embryonic day (E)13.5, the cyt1 isoform is selectively expressed by migrating cINs entering the dorsal pallium, not in those still in the ganglionic eminences, and it seems critical for their ability to traverse the pallial-subpallial boundary (Rakić et al., 2015). Furthermore, Cdk5 positively regulates the ErbB4/PI3-kinase/AKT pathway by phosphorylating ErbB4 (Rakić et al., 2015). Thus, altering of ErbB4 signaling through defective Cdk5 phosphorylation, PI3-kinase binding or alterations in both molecular pathways impair leading process morphology, directionality and polarity of MGE-derived INs, as well as their ability to enter the dorsal pallium (Rakić et al., 2015). This cascade is clinically relevant since disruption of Nrg1-ErbB4 signaling is associated with epilepsy, intellectual disability and schizophrenia (Li et al., 2011; Tan et al., 2011; Marin, 2012; Del Pino et al., 2013; Hyder et al., 2021).

### 2.3. Integration into migratory streams, intracortical dispersion, and laminar allocation

Once they cross the pallial-subpallial boundary, cINs integrate migratory streams on either side of the cortical plate. In early stages of cortical development (E12-E13), most interneurons migrate towards the developing cortex via two parallel routes, a superficial migratory stream (SMS) that passes through the marginal zone (Bastaki et al., 2017). and a deep migratory stream (DMS) localized in the subventricular zone (SVZ). Between E15 and E16 in mice, a third migratory stream appears in the subplate (SP) between the MZ and the SVZ streams (Lavdas et al., 1999; Wichterle et al., 2001; Marin, 2013; Peyre et al., 2015). Although earlier evidence suggested that migratory route allocation is independent of an interneuron’s birthplace (Miyoshi and Fishell, 2011), recent evidence suggests that cell identity may actually determine, at least in part, the chosen migratory stream. Indeed, somatostatin-expressing Martinotti cells as well as translaminar parvalbumin-expressing cells preferentially migrate through the MZ (Lim et al., 2018). Interestingly, these interneurons send axonal projections to cortical layer I post-natally, a region arising from the MZ. Hence, integration into a migratory stream seems linked to cell fate (early specification) and may contribute to axonal targeting (Lim et al., 2018). It is thus likely that cINs migrate through the MZ or SVZ depending on their response to different extracellular guidance cues, although the identity of these signals as well as the underlying mechanisms remain largely unknown. Transcriptomics data from migrating cINs showed differential gene expression profiles between cINs migrating within the superficial or deep migratory streams, including different sets of guidance receptors (Antypa et al., 2011). Further, a recent study demonstrated that EphB2/EphrinA5 signaling maintains the segregation of the SVZ and SP migratory routes. EphrinA5, highly expressed in the deep ventricular zone (VZ), upper SVZ, deep intermediate zone (IZ) and the CP of the developing telencephalon, confines EphB2-expressing interneurons to the SVZ and SP streams through a repulsive effect (Liu et al., 2017). Similarly, fibronectin leucine-rich transmembrane proteins (FLRT2 and 3), expressed by pyramidal cells and previously known for their roles in axon guidance, excitatory neuron migration and synaptogenesis (Yamagishi et al., 2011; O'Sullivan et al., 2012; Leyva-Díaz et al., 2014; Del Toro et al., 2017, 2020), have recently been shown to exert repulsive effects on Unc5B/D-expressing cINs *in vitro* (Fleitas et al., 2021). *In vivo*, these repulsive cues cooperate to maintain the integrity of the SP stream, as the loss of both FLRT2 and 3 in pyramidal cells affects the cortical distribution of cINs. In *Flrt2/3* double knockout mouse model, cINs normally found in the SP stream abnormally accumulate in the IZ, while the organization of the SVZ stream remains intact (Fleitas et al., 2021).

Neurotransmitter signaling can also modulate cIN migration. *In vivo*, mice lacking the glycine receptor α2 subunit homomers specifically in cINs show migration defects in the SVZ, but not the MZ or SP streams (Avila et al., 2013). Furthermore, pharmacological blockade of GABA_B_ receptors leads to an aberrant accumulation of cINs in the SVZ stream and a decrease in the MZ stream, suggesting that GABA signaling is also important for migratory route selection (Lopez-Bendito et al., 2003).

Intracortical dispersion involves the timed exit from migratory streams and a switch of migration modes from tangential to radial migration. Chemokine Cxcl12 (previously known as Sdf-1), expressed by meningeal and progenitor cells in the SVZ (Stumm et al., 2003; Tiveron et al., 2006) exerts a dual role in interneuron migration, confining migrating cINs to the migratory streams and controlling the timing of CP invasion. Its function in migrating cINs is mediated by two G protein-coupled receptors, Cxcr4 and Cxcr7, both essential for proper sensing of this chemokine. Indeed, the absence of either receptor leads to the premature departure of cINs from the migratory streams and their precocious invasion of the CP, perturbing cIN laminar positioning in the postnatal cortex (Li et al., 2008; López-Bendito et al., 2008; Tanaka et al., 2010; Wang et al., 2011). These findings suggest that the CP exerts a chemoattractive effect on migrating cINs. Interestingly, Cxcl12 reduces the branching dynamics of cIN leading process through the regulation of actin and microtubules (Lysko et al., 2011, 2014), thus decreasing the ability of cINs to sense short-range environmental cues present at significant distance from the tangential migratory streams. It was later discovered that the developing CP is highly enriched in neuregulin-3 (Nrg3), a short-range chemoattractant expressed by pyramidal cells. The *NRG3* gene has been linked to schizophrenia in human genetic studies and *Nrg3* knockout mice display behavioral deficits mirroring those observed in patients (Meier et al., 2013; Hayes et al., 2016). *In vitro*, tangentially migrating MGE-derived INs are attracted by both Cxcl12 and Nrg3, but they display a preference for Cxcl12. However, overexpressing Nrg3 hastens the invasion of the CP by MGE-derived INs expressing the receptor ErbB4. These experiments suggest that the timed invasion of the CP, which is essential for the proper lamination of cINs, depends on the fine-tuned balance between Cxcl12 and Nrg3 (Bartolini et al., 2017).

Moreover, disrupting the fate of cortical pyramidal cells changes the laminar distribution of cINs, suggesting that pyramidal cells instruct cIN positioning through specific guidance cues. For instance, deep layer pyramidal tract neurons, which typically project to the thalamus, brainstem and spinal cord, inform the positioning of MGE-derived cINs (the parvalbumin-and the somatostatin-expressing cINs) in cortical layer V. Thus, the deletion of *Fezf2*, inducing a fate-switch from subcerebral projection neurons towards callosal projection neurons, results in massive reduction of MGE-derived cINs in layer V (Lodato et al., 2011). In explants and *in vivo*, cortical pyramidal cells specifically attract cINs that would typically target them, such that deep-layer corticofugal pyramidal neurons tend to attract early-born MGE-neurons while callosal projecting pyramidal neurons attract later-born cINs (Lodato et al., 2011). Notably, the subtype of PC and cIN seems more important for their proper pairing than their chronological appearance (Lodato et al., 2011). Similarly, the deletion of *Satb2* to reprogram intratelencephalic pyramidal neurons that usually project to other cortical areas and the striatum into pyramidal tract neurons projecting to subcortical structures selectively disrupts the lamination of CGE-derived INs (Wester et al., 2019). Thus, distinct populations of cortical projection neurons might control the lamination of cINs, likely through their release of specific cues, which must be further be identified.

Interestingly, cINs interact with other cIN populations and this crosstalk also regulates the final distribution of specific cIN populations. For instance, MGE-derived cINs, which populate deep cortical layers, secrete semaphorin 3A that repulses PlexinA4 receptor-expressing CGE-derived INs, ultimately confining CGE-INs to superficial cortical layers as they enter the cortical plate (Limoni et al., 2021).

Emerging neuronal network activity also plays an essential role in cIN development and migration (Zimmer-Bensch, 2018). For instance, before postnatal day (P)3 when radial migration is ongoing, a decrease in neuronal excitability through overexpression of the inward rectifier potassium channel Kir2.1 in CGE-derived INs leads to a shift in the cortical distribution of calretinin-positive and reelin-positive cells, but not VIP-positive cells, from superficial to deep cortical layers (De Marco Garcia et al., 2011). Interestingly, the level of activity after P3 regulates the morphology, but not the positioning, of these same cell types (De Marco Garcia et al., 2011). Furthermore, the source of input seems critical to regulate these processes. For instance, glutamatergic inputs from the thalamus specifically regulates the morphological maturation of reelin-expressing INs, without affecting VIP-positive cells, while manipulating cortical glutamatergic inputs does not affect the morphology of either cell types (De Marco Garcia et al., 2015). Thus, distinct subtypes of cINs might rely on specific sources of neuronal activity for their development, migration, and maturation in cortical circuits.

### 2.4. Termination of interneuron migration

Once settled in the appropriate cortical layers, cINs must stop their migratory behavior. In mice, this phenomenon occurs during the first postnatal week (Bortone and Polleux, 2009). It was first suggested that migrating cINs perceive GABA as a stop signal during early postnatal development, when the expression of potassium/chloride exchanger KCC2 is upregulated in these cells (Bortone and Polleux, 2009). KCC2 mediates the inversion of the intracellular chloride gradient. Consequently, GABA becomes hyperpolarizing and, through its activation of GABA_A_ receptors, decreases the frequency of intracellular calcium transients and slows IN motility (Bortone and Polleux, 2009). Although it was initially proposed that KCC2 expression suffices to trigger the arrest of cIN migration, it was recently demonstrated that the lack of KCC2 in cINs does not alter their ability to migrate to their final cortical allocation, arguing that other molecular actors might determine the arrest of cIN migration (Zavalin et al., 2022). Moreover, reduction of cIN motility is also observed when migrating cINs are co-cultured with postnatal cortical cells, suggesting that unknown extrinsic cues secreted by cortical cells might also act as stop signals for migrating cINs through yet elusive mechanisms (Inamura et al., 2012).

## 3. Blood vessels as a source of guidance cues for migrating interneurons

Several recent studies have demonstrated that the development of the vascular system is crucial for many aspects of cortical maturation, including neuronal migration (Paredes et al., 2018). Two distinct types of vascular structures are found in the embryonic brain: the periventricular vascular plexus (PVP) and the pial network, also known as perineural vascular plexus (PNP; see Figure 1B). These two blood vessel systems are different in their anatomical location, developmental timeline, and gene expression (Vasudevan and Bhide, 2008). The PNP is generated by the neural tube and covers the pial surface of the cortex around embryonic day 10 (E10) in mice (Hogan et al., 2004). In comparison, PVP formation begins at E11 following a ventro-dorsal angiogenic gradient, aligned with the future direction of cINs migration starting a day later (Vasudevan et al., 2008). The PVP was recently shown to regulate neurogenesis and the generation of MGE-derived cINs (Tan et al., 2016). Migration of MGE-derived INs in mice starts at E13.5 (Lavdas et al., 1999; Marin and Rubenstein, 2001), corresponding roughly to humans IN migration that has been shown to be in progress during the late stage of gestation (Xu et al., 2011). Both in mice and humans, vascular development slightly precedes the onset of IN migration, suggesting a potential role for early brain vascular structures in instructing migrating INs. Moreover, these two vascular structures are in close proximity to the two migratory routes followed by cINs, the PNP lining the edge of the SMS, and the PVP closely aligned with the DMS (see Figure 1B; Won et al., 2013). This spatial proximity as well as the temporal coincidence of PVP development with cIN migration suggests opportunities for potential interactions between the developing brain vasculature and migrating cINs, as detailed below.

### 3.1. Vascular-neuronal interactions and the roles of endothelial cells in guiding cINs migration

Endothelial cells (ECs) from cortical blood vessels impact neocortex formation by secreting molecular cues that influence neuronal cell behavior (Karakatsani et al., 2019). Interestingly, the MGE becomes actively vascularized in the days preceding the initiation of MGE-IN migration, suggesting that vessels in the MGE may secrete cues that will help initiate cIN migration. The work of Genestine et al. (2021) helped identify two EC-derived paracrine factors released in the MGE, SPARC and SerpinE1, which promote the tangential migration of MGE-derived cINs in mice MGE explants and organotypic slice cultures at E11.5, and also favour cIN migration from human stem cell derived organoïds. SPARC protein has been previously shown to be implicated in multiple different cellular events, such as migration of malignant cells (Arnold and Brekken, 2009). SerpinE1, on the other hand, is implicated in the uPA/urokinase pathway (Mahmood et al., 2018), which plays a known role in IN tangential migration (Powell et al., 2001). In addition to their intracellular contribution, these proteins likely also participate in IN migration by reducing the cell adherence to the extracellular matrix (Gongidi et al., 2004). SPARC and SerpinE1 are enriched in brain ECs compared to the rest of the brain and to EC of other organs (Hupe et al., 2017). Notably, Genestine et al. (2021) showed that the inactivation of either SPARC or SerpinE1 using antibody-mediated interference reduces the ability of MGE-derived medium to stimulate cIN migration *in vitro*, and that both proteins likely act in a complementary fashion within the same molecular pathway.

In addition, the vascular endothelial growth factor Vegfa, a pro-angiogenic factor expressed by ECs and neural progenitors and critical for the formation of the brain’s vasculature (Ruhrberg et al., 2002), also appears to play a critical role in cIN migration (Haigh et al., 2003; Raab et al., 2004). Vegfa exists in three isoforms, Vegfa120, Vegfa165, and Vegfa188, differing in their expression of a heparan sulfate proteoglycan binding domain and their ability to bind the extracellular matrix. Vegfa ablation was shown to reduce the number of cINs (Li et al., 2013), partly by impairing the expression of *Dlx1/2*, a transcription factor required for IN specification and migration (Darland et al., 2011; Cain et al., 2014). Using a mouse model carrying a deletion of the Vegfa165/188 isoforms and ubiquitously expressing the Vegfa120 isoform, circumventing the early lethality of pan Vegfa knockout models, Barber et al. (2018) found that cINs populate the cortex at mid-gestation, despite aberrant brain vascularization and angiogenesis, although migration of cINs in late gestation is greatly impaired, resulting in a net reduction of cIN numbers at birth, with altered distribution and proximity to developing vessels (Barber et al., 2018).

Early during cIN migration, both GABA and glutamate act as motogenic factors that promote cIN migration (Bortone and Polleux, 2009). Furthermore, cINs require the functional expression of GABA_A_ receptor subunits to ensure their tangential migration (Cuzon Carlson and Yeh, 2011). However, the exact source of GABA that triggers this effect was unclear. Li et al. (2018) recently demonstrated that endothelial derived GABA is essential for this process. First, endothelial cells require functional GABA_A_ receptors and a GABA release mechanism (*Vgat*) for appropriate brain angiogenesis Li et al. (2018). Secondly, GABA release from PVP blood vessels promotes cIN migration (Li et al., 2018), while neuronal-derived GABA could not compensate for the reduced (*Gabrb3^ECKO^*) or null (*Vgat^ECKO^*) release of endothelial cell-derived GABA in ensuring these processes (Li et al., 2018). Nonetheless, further research is required to determine how, precisely, GABA influence IN migration at the cellular and molecular levels.

Glutamate also influences the interaction between the vascular network and migrating cINs. Recent findings suggest that ECs NMDA receptor subunits expression is region-specific and coincides with late cIN migration (Legros et al., 2009; Luhmann et al., 2015). Glutamate activation of endothelial NMDA receptors leads to the recruitment of two proteases, matrix metalloproteinase-9 (MMP-9) and tissue-plasminogen activator (t-PA), who in turn increase cIN migratory speed alongside the PNP and radial-microvessels in the mouse superficial cortex (Léger et al., 2020). Medications altering this process, such as NMDA antagonist anesthetics, could thus potentially interrupt the process of late cIN migration in neonates at a time when a portion of cINs are still migrating along radial microvessels, warranting caution at this age (Xu et al., 2011; Léger et al., 2020). Subsequent studies at a subcellular level are needed to address how theses proteases are able to promote cIN migration speed.

### 3.2. Crosstalk between blood vessels, glial cells and migrating interneurons

Recent discoveries in the field of vascular guidance of cIN migration revealed the critical role of ventrally-derived oligodendrocyte precursor cells (vOPCs). First-wave vOPCs undergo substantial cell death shortly after birth. Their contribution to cIN development was thus unclear (Kessaris et al., 2006). However, Lepiemme et al. (2022) recently described significant contributions of these vOPCs to the guidance of cIN tangential migration. Both cINs and vOPCs emerge from common embryonic origins (MGE, POA) and follow parallel migratory routes, responding to the chemoattractant Cxcl12 at the level of the cortical plate (Lepiemme et al., 2022). However, despite these similarities, there is minimal overlap between cINs and vOPCs migratory streams. While vOPCs migrate along the cortical blood vessels expressing Cxcl12, cINs remain in organized streams within the parenchyma (Tsai et al., 2016; Lepiemme et al., 2022). Upon depletion of first-wave vOPCs, cINs start to cluster around Cxcl12-expressing blood vessels and halt their migration (Lepiemme et al., 2022). vOPCs thus prevent migrating cINs from aggregating to the Cxcl12-enriched blood vessels through a unilateral contact repulsion (UCoRe) mechanism (Lepiemme et al., 2022). Importantly, this critical function cannot be performed by second-wave vOPCs, which fail to rescue the cINs migration deficit in mutants devoid of first wave vOPCs, suggesting either age-specific mechanisms in earlier born cINs or first-wave vOPC-specific signaling molecules (Lepiemme et al., 2022).

## 4. Cell-intrinsic regulation of cIN migration dynamics

### 4.1. Transcriptional regulation of cIN migration

**Figure 2:**
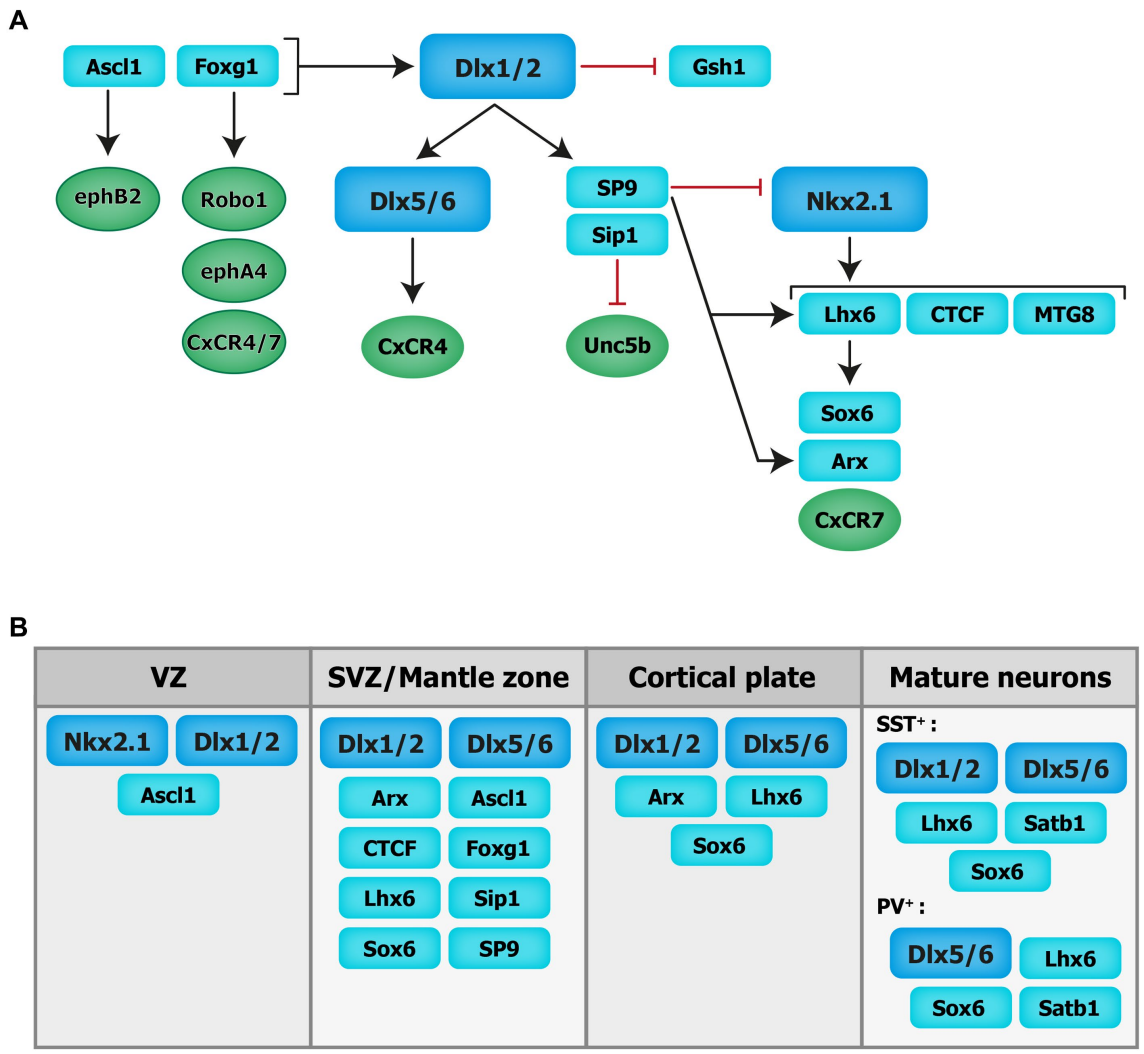
Transcriptional regulation of cortical interneuron migration. Schematics of the molecular cascade that regulates cIN migration. Several transcription factors contribute to the regulation of cIN migration, in part by controlling the expression of key guidance receptors (in circles). VZ: ventricular zone. SVZ: subventricular zone.

cIN migration is under the control of both extracellular signals and cell-autonomous intrinsic programs. Transcription factors, in addition to their fundamental roles in cell specification and differentiation, regulate cIN migration in part by controlling the expression of critical receptors and downstream molecular signaling cascades. During forebrain development, the generation of MGE-derived INs relies on the expression of several TFs including the *Dlx* homeobox genes *Dlx1/2* and *Dlx5/6*, the *NK2* homeobox 1 gene (*Nkx2-1*) and the *LIM* homeobox protein 6 [*Lhx-6*; see reviews (Bandler et al., 2017; Hu et al., 2017; Christodoulou et al., 2022; see Figure 2)].

The distal-less (*Dlx*) homeobox genes *Dlx1/2* and *Dlx5/6* are at the core of the genetic cascade controlling prenatal and postnatal IN development (Wang et al., 2010; Le et al., 2017; Pla et al., 2018). In *Dlx1/2* knockout mice, which die at birth, cINs fail to migrate out of the ganglionic eminences, resulting in a reduction of IN numbers in the cortex and hippocampus (Anderson et al., 1997). These migratory deficits were rescued upon overexpression of *Arx* or by decreasing the levels of *Gsh1*, suggesting that these downstream transcription factors are necessary for mediating *Dlx*-dependent regulation of IN migration (Colasante et al., 2008; Wang et al., 2013).

Moreover, *Dlx1/2* promotes the subpallial expression of the *Sip1* transcription factor (McKinsey et al., 2013). Notably, the deletion of *Sip1* from MGE-derived INs severely comprises their migration and maturation (van den Berghe et al., 2013). This is largely due to an upregulation of the guidance receptor Unc5b in *Sip* knockout INs, which misguides migrating INs towards ventral regions away from the cortex. Thus, downregulating Unc5b rescues the cIN migratory deficit in *Sip1* mutants (van den Berghe et al., 2013), suggesting that *Sip1* acts as a critical regulator of cIN migration by controlling Unc5b expression in a cell-autonomous manner (van den Berghe et al., 2013).

Upstream of *Dlx1/2* is the Achaete-scute family bHLH transcription factor (*Ascl1*) and Forkhead box G1 transcription factor (*Foxg1*; Poitras et al., 2007; Yang et al., 2017). *In utero* electroporation of both *Ascl1* and *Dlx2* in the dorsal telencephalon of mice promotes tangential migration along the SVZ/VZ and IZ. Further, knockdown of *Dlx2* and overexpression of *Ascl1* leads to a reduction in the number of INs migrating through the SVZ/VZ, but increases the number of INs migrating through the IZ (Liu et al., 2017). Moreover, chromatin immunoprecipitation assays confirmed that EphB2 receptor, which binds to the repulsive molecule EphrinA5, is a direct downstream target of *Ascl1*. Disruption of EphB2/EphrinA5 signaling alters tangential migration as INs fail to confine to the DMS (Liu et al., 2017). These results indicate that *Ascl1* promotes tangential migration in two distinct ways: through the induction of *Dlx2* expression and in a *Dlx2*-independent fashion through the induction of *Ephb2* expression.

*Foxg1*, encoding a transcription factor associated with autism, Rett syndrome, epilepsy and intellectual disability (Seltzer et al., 2014; Wong et al., 2019; Miyoshi et al., 2021), acts upstream of *Dlx1/2* and *Ascl1* to regulate their expression levels and control IN migration (Yang et al., 2017). Indeed, conditional deletion of *Foxg1* in the SVZ and mantle zone of the MGE at E13.5 impairs the formation of tangential migratory streams. At E18.5, both MGE- and CGE-derived INs fail to reach the cortex and abnormally accumulate in the subpallium. Loss of *Foxg1* in migrating INs led to morphological defects (shorter neurites and decreased branching). Additionally, several receptors required for proper guidance of migrating INs, such as *Robo1, EphA4* and *Cxcr4/7*, were significantly downregulated in absence of *Foxg1* (Yang et al., 2017).

*Nkx2-1,* expressed in the MGE and POA, maintains regional identity by repressing other transcription factors in adjacent embryonic regions and initiates, through a permissive chromatin state, the expression in SVZ and mantle zone progenitors of transcription factors which regulate MGE-derived lineages (Sandberg et al., 2016). A direct downstream target of *Nkx2-1* is *Lhx6* (Liodis et al., 2007), whose expression starts around E11.5 and persists during IN migration and maturation, suggesting that it plays roles beyond IN specification. *Lhx6* null mutant display significant delays in MGE-derived cIN migration resulting in severe reductions and abnormal positioning of somatostatin- and parvalbumin-expressing INs in superficial and deep cortical layers in the post-natal cortex (Liodis et al., 2007). Similar phenotypes were seen when the Sry-related HMG box transcription factor *Sox6*, acting downstream of *Lhx6*, was conditionally ablated from MGE cells (Batista-Brito et al., 2009). However, in *Lhx6* null mutants, transduction of mutant MGE cells with viruses expressing *Sox6* did not rescue the cellular and laminar phenotypes of these mice, suggesting that other molecular mechanisms are at play (Vogt et al., 2014). Notably, chromatin immunoprecipitation experiments revealed that *Lhx6* directly binds enhancers near the Aristaless-related homeobox transcription factor (*Arx*) and *CXCR7* gene locus, therefore promoting their expression. Interestingly, transduction of *Lhx6* mutant MGE cells with viruses expressing *Arx* or *CXCR7* rescues the expression of somatostatin and parvalbumin and the laminar distribution of cINs, respectively, suggesting that *Arx* activity is important for IN differentiation while *CXCR7* plays a key role in laminar allocation, in addition to its roles in tangential and radial migration (discussed above; Vogt et al., 2014).

Myeloid translocation gene 8 (MTG8) is a non-DNA binding transcriptional regulator expressed in the SVZ of the MGE, CGE and LGE at an early embryonic stage (E11.5), with progressive broader expression in the cortical plate and PC progenitors (E16.5; Asgarian et al., 2022). During early embryonic stages MTG8 interacts with *Lhx6* in MGE-derived cIN to specifically promote somatostatin- NPY-expressing cINs cell-fate before the onset of migration (Asgarian et al., 2022). Other regulators of *Lhx6* expression, acting upstream in the molecular cascade, include the zinc-finger CCCTC-binding factor (CTCF), acting as a regulator of chromatin organization (Merkenschlager and Nora, 2016). Recently, *de novo* mutations in the *CTCF* gene were associated with ASD, microcephaly, schizophrenia, and intellectual disability (Gregor et al., 2013; Iossifov et al., 2014; Juraeva et al., 2014; Bastaki et al., 2017). Conditional inactivation of *Ctcf* in early neural progenitors reduces the expression of *Lhx6* and a few of its downstream effectors, including *Sst* and *Cxcr4*, while *Nkx2-1* transcript levels remain unchanged. This reduction in *Lhx6* transcripts is associated with a delay in tangential migration prenatally (possibly due to the loss of Cxcr4) as well as a significant reduction in the number of somatostatin- and parvalbumin-expressing INs in the postnatal cortex along with lamination defects (Elbert et al., 2019). Notably, the re-expression of *Lhx6* in CTCF-null MGE cells rescues the number of somatostatin-expressing INs but not parvalbumin-expressing INs (Elbert et al., 2019). Further, recent transcriptomics data have shown zinc-finger transcription factor *Sp9*, expressed in the ganglionic eminences (Zhang et al., 2016b), as an upstream regulator of the transcription factors *Lhx6*, *Nkx2-1*, *Arx* and *Zeb2*. In mice, the conditional loss of *Sp9* in MGE-derived INs leads to disorganized migratory streams, with more cells in the DMS vs. the SMS and an abnormal increase in the ratio of somatostatin- vs. parvalbumin-expressing cells. At the molecular level, *Lhx6*, *CXCR7* and *Arx* transcripts levels are significantly reduced in the mutant mouse compared to controls at different developmental timepoints (E12.5, E13.5, and E15.5; Liu et al., 2019). Interestingly, *Sp8* is significantly upregulated in the MGE of *Sp9* mutant mice, offering some degree of functional compensation, whereas the combined deletion of *Sp8* and *Sp9* results in greater defects in MGE-derived cINs numbers and distribution (Tao et al., 2019).

### 4.2. Cytoskeletal reorganization during migration

From a cellular point of view, cINs migration is a cyclic process comprising three stages during which the morphology of cINs dynamically changes to allow saltatory movement. During the first step, cINs elongate their leading process and extend several branches to sense the surrounding environment through filopodia and lamellipodia. Once a branch stabilizes in a specific direction, a swelling containing the centrosome and Golgi apparatus advances toward the leading process. The second step involves nucleokinesis, which consists of a fast anterograde nuclear translocation into the swelling. Lastly, in most cases, cINs retract their trailing process (Bellion et al., 2005; Lepiemme et al., 2020). Each step is controlled by the rearrangements of the cytoskeleton in response to extracellular and intracellular signals. The cytoskeleton is mainly composed of two major components: actin filaments (F-actin) and microtubules (MT). F-actin results from the polymerization of small globular proteins called g-actin. F-actin is highly dynamic and continuously assembles and disassembles, preferentially at the barbed (+) end (Lehtimäki et al., 2017).

Among the proteins that regulate actin filaments, we find the non-muscle myosin II that forms actomyosin networks. During migration, INs are pulled forward by contractile forces generated by the actomyosin network (Bellion et al., 2005; Martini et al., 2009; Martini and Valdeolmillos, 2010). Multiple factors acting on the actomyosin network in migrating cINs have been described. Elongator is a protein complex composed of six subunits including the Elp1 scaffold subunits and the Elp3 enzymatic core (Li et al., 2001; Winkler et al., 2001). Elongator is implicated in several processes such as the control of mRNA translation efficiency (Nedialkova and Leidel, 2015) and paternal genome demethylation (Okada et al., 2010). Pathogenic variants in Elongator subunits have been associated with moderate to severe neurodevelopmental disorders (Toral-Lopez et al., 2020; Duan et al., 2021; Kojic et al., 2021; Gaik et al., 2022; Kojic et al., 2023). Elongator promotes cIN tangential migration during corticogenesis by regulating nucleokinesis and the dynamics of leading process branching (Tielens et al., 2016). In the normal state, non-phosphorylated (active) cofilin induces a depolymerization of actin filaments into globular actin. A balance between the phosphorylated (inactive) and non-phosphorylated (active) form is typically maintained. Cofilin, together with Myosin II activated by the phosphorylation of the myosin light chain (MLC), regulates the nucleokinesis and branching of cINs leading process. However, the conditional deletion of the *Elp3* subunit in newborn cINs reduces cofilin phosphorylation and increases MLC phosphorylation in the soma and growth cone of migrating cINs, resulting in altered actin cytoskeletal reorganization and reduced actomyosin contractility, impairing nucleokinesis and branching (Tielens et al., 2016).

MT, the second component of the cytoskeleton, are composed of β-tubulin and α-tubulin, located, respectively, at their plus and minus ends (Janke and Magiera, 2020). The centrosome acts as a major assembly points for MT, with assembly and disassembly occurring at MT plus ends (Garcin and Straube, 2019). MT dynamics are crucial to all neuronal development steps including migration (Kuijpers and Hoogenraad, 2011). During cIN migration, MT form a cage-like structure around the nucleus that facilitates nuclear translocation (Godin et al., 2012). MT are also required for the extension of the leading process (Godin et al., 2012). Several microtubule-associated proteins (MAPs), including the doublecortin (DCX) and Lissencephaly-1 (Lis-1) proteins, participate in the organization and function of MT and are key regulators of pyramidal cell radial migration. Their loss results in brain malformations known as lissencephaly, characterized by altered lamination and gyration of the neocortex, resulting in developmental delay, intellectual disability and sometimes epilepsy (Matsumoto et al., 2001; Reiner and Sapir, 2013). Both DCX and Lis-1 have been shown to play critical roles in cIN migration, likely contributing to the global clinical manifestations of these disorders. DCX stabilizes and bundles MT within the leading process and regulates the interaction between MT and actin. Accordingly, cINs lacking DCX display MT instability that results in excessively branched leading processes (Lysko et al., 2014). Lis-1 regulates dynein, a cytoplasmic motor protein implicated in the transport of vesicles towards the minus ends of MT (Roberts et al., 2013). Loss of *Lis-1* in cINs alters their tangential migration (McManus et al., 2004). p27^kip1^ is another MAP implicated in the coordination of both MT network and actomyosin contractility. The conditional deletion of *p27^kip1^* impacts cIN migration through an overactivation of myosin II (Godin et al., 2012).

MT are subject to several posttranslational modifications that regulate their biological functions, including during cell migration (van Dijk et al., 2008; Creppe et al., 2009; Hahn et al., 2013; Tanco et al., 2013). Polyglutamylation adds a peptide chain of glutamate to the target protein by enzymes known as polyglutamylases (ex: TTLL1; Janke et al., 2005; Janke and Kneussel, 2010). As this modification is reversible, the glutamate chain is removed by a cytosolic carboxypeptidase (CCP) enzyme (van Dijk et al., 2007). Pathogenic variants of *CCP1* have been associated with developmental delay (Firth et al., 2009). *Ccp1* mRNA is highly expressed in the developing subpallium (Silva et al., 2018). The conditional loss of *Ccp1* from post-mitotic cINs impairs cytoskeletal remodelling and actomyosin dynamics, leading to a decrease in the amplitude of nucleokinesis and reduced pause duration, without affecting speed (Silva et al., 2018). Notably, whereas actomyosin contraction is usually polarised at the rear of the nucleus to push the nucleus forward, *Ccp1* cKO cINs display a failure of actomyosin polarization resulting in a switch from saltatory migration to a “treadmill-like motion” (Silva et al., 2018). This abnormal phenotype is due to the aberrant enzymatic regulation of MLCK in the absence of CCP1 by mechanisms dependent of its function on MT depolyglutamylation (Silva et al., 2018). Interestingly, this reduction of pause duration dominates the phenotype, such that the loss of *Ccp1* ultimately leads to a net increase of cIN invasion in the cortical plate while also enhancing the proliferation of intermediate progenitors giving rise to upper layer projection neurons in the dorsal pallium, ultimately altering the balance of excitation and inhibition in the cortex (Silva et al., 2018).

Microtubule-actin cross-linking factor 1 (MACF1) is a member of the ubiquitous plakin family of cytoskeletal linker proteins (Suozzi et al., 2012). By coordinating the organization of both MT and actin filaments, MACF1 is implicated in many cellular processes such as axonal growth and cell migration (Goryunov and Liem, 2016). Recently, *MACF1* mutations have been associated with lissencephaly, severe intellectual disability and epilepsy (Dobyns et al., 2018). MACF1 has several isoforms, some of which are highly expressed in the brain. At E12.5, MACF1 is enriched in the ventricular zone, whereas it becomes mostly expressed in the cortical plate at E15.5 (Ka et al., 2017). MACF1 regulates the migration of pyramidal cells (Ka et al., 2014) and cINs (Ka et al., 2017). Indeed, the conditional deletion of *Macf1* in mouse cINs progenitors leads to a reduction and an abnormal distribution of cINs, which accumulate in the intermediate zone during migration and largely fail to populate the dorsal pallium (neocortex and hippocampus), due to a premature switch from tangential to radial migration (Ka et al., 2017). The loss of *Macf1* also impacts the morphology of cINs by promoting aberrantly complex neurites, which are shorter and more branched, largely due to a defect in MT stabilization in *Macf1* knockout INs (Ka et al., 2017). This reflects the role of MACF1 as an actin-MT linker that coordinates MT dynamics (Kodama et al., 2003).

### 4.3. Rho GTPases and their regulators and effectors involved in cIN migration

**Figure 3:**
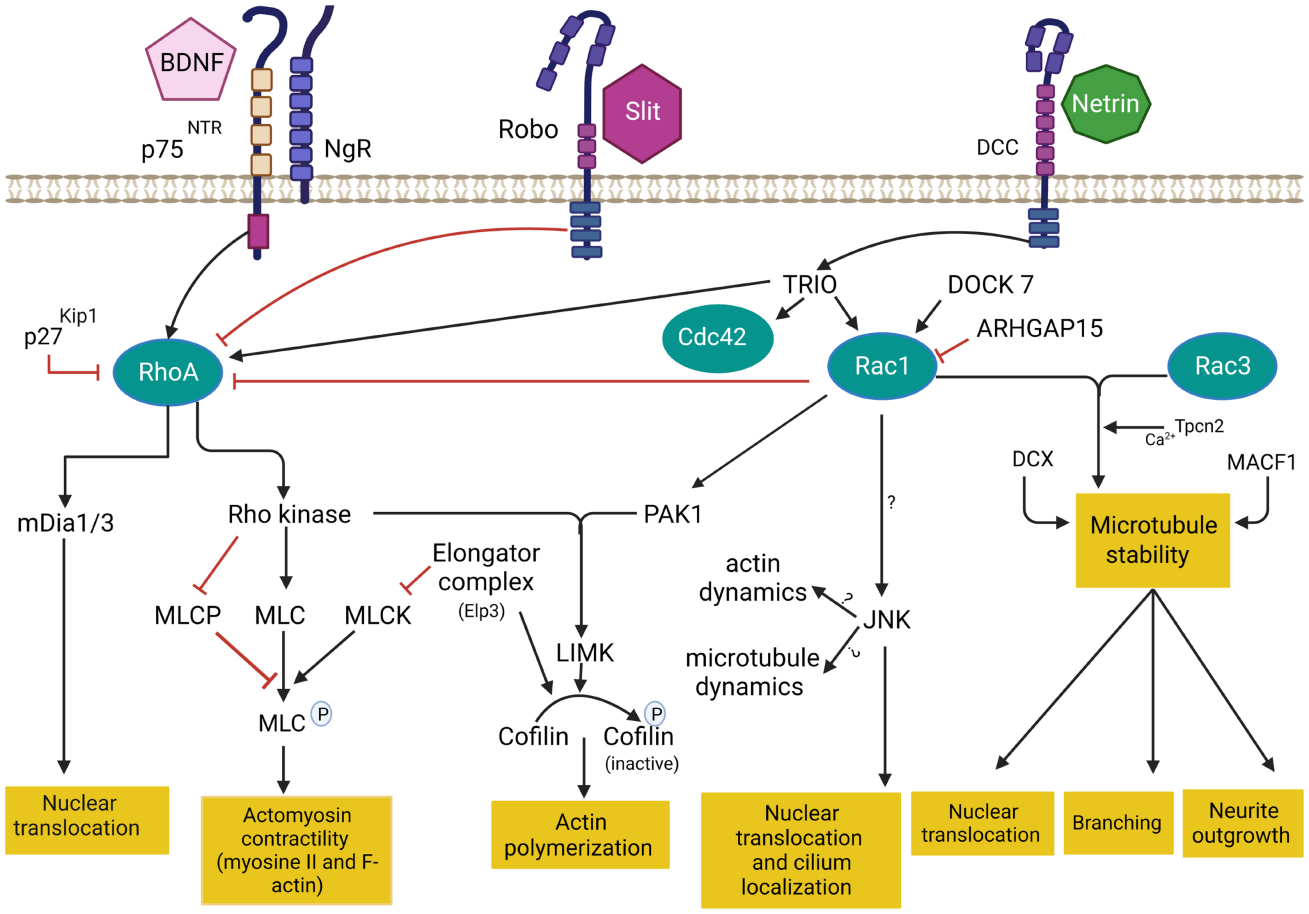
Cytoskeleton remodeling during IN migration is regulated by extrinsic cues and intracellular molecular cascades that relay these signals to the cytoskeleton. Various intracellular molecular cascades signal from the cell membrane to the actin and microtubule cytoskeleton. Rho GTPases are key regulators of these processes. This figure was created using BioRender.com. DCC: deleted in colorectal cancer. BDNF: Brain-derived neurotrophic factor. NgR: Nogo-66-Receptor. P75^NTR^: The neurotrophin receptor p75. Rac1: ras related C3 botulinum toxin substrate 1. RhoA: ras homologous member A. Cdc42: cell division cycle 42. mDia: diaphanous-related formin. MLC: myosin light chain. MLCP: myosin light chain phosphorylated. MLCK: myosin light chain kinase. PAK1: p21-activated serine/threonine kinase 1. LIMK: lim domains kinase. JNK: c-jun N-terminal kinase. DCX: doublecortin protein. MACF1: Microtubule-actin cross-linking factor 1. Tpcn1: two pore segment channel 2. DOCK7: (Dedicator of cytokenesis7). ARHGAP15: GTPase-activating protein 15. TRIO: Trio Rho Guanine Nucleotide Exchange Factor.

Genomic studies in patients with autism spectrum disorders, epilepsy or intellectual deficiency have identified pathogenic mutations in multiple Rho GTPases-encoding genes, but also in various Rho GTPase regulators and effectors (*RAC1, CDC42, PAK*; Michaud et al., 2014; Tastet et al., 2019; Barbosa et al., 2020; Halder et al., 2022; Dobrigna et al., 2023). Deregulation of Rho GTPases thus seems to be a shared molecular mechanism between several monogenic forms of neurodevelopmental disorders. Interestingly, recent evidence suggests that Rho GTPases, as well as their regulators and effectors, play essential roles during IN migration by coordinating cytoskeletal dynamics. Small GTPases of the Rho classes (Rho GTPases) are highly conserved signal transducing enzymes that switch between GTP and GDP-bound states in response to stimuli (Bos et al., 2007). The GTPases are highly regulated by the Rho guanine nucleotide exchange factors (RhoGEFs) and Rho GTPase activating proteins (RhoGAP). RhoGEF activate GTPases by promoting the switch from an inactive GDP-bound conformation to an active GTP-bound conformation, while RhoGAP downregulate GTPase signaling by enhancing the intrinsic GTP hydrolysis activity of GTPases (Rossman et al., 2005; Hodge and Ridley, 2016). Rho GTPases integrate different extracellular and intracellular cues to reorganize the actin cytoskeleton and are critical in several cellular aspects of brain development, including neuronal migration, axonal guidance, and synaptic plasticity (Govek et al., 2005; Ba et al., 2013; Cannet et al., 2014; Herring and Nicoll, 2016; Gentile et al., 2022; see Figure 3).

The best-characterized members of the Rho family in the brain are Rac1 (ras related C3 botulinum toxin substrate 1), RhoA (ras homologous member A) and Cdc42 (cell division cycle 42; Azzarelli et al., 2014) Rac1 plays a key role in the formation of lamellipodia and membrane protrusion at the front of migrating cells (Bisaria et al., 2020). RhoA regulates cell retraction during migration and induces the formation of actin stress fiber via Rho-associated protein kinase (Riedl et al., 2008 that phosphorylates myosin light chain (MLC) required for myosin II activation. RhoA also activates diaphanous-related formin mDia1/2 to control cell retraction (Watanabe et al., 1999). Cdc42 induces the formation of filopodia in the leading edge through the activation of mDia2 (Peng et al., 2003). Cdc42 and Rac1 activate Wasp/WAVE complex, which in turn activates Arp2/3 protein (Takenawa and Suetsugu, 2007). During leading process branching, Arp2/3 together with contractin allow the formation of new membrane protrusions by the assembly of F-actin. These protrusions are then stabilized by MT and support the formation of new branches (Martini et al., 2009; Spillane et al., 2011; Lysko et al., 2014; Peyre et al., 2015). In addition, Wasp/WAVE complex also activate ROCK which phosphorylates profilin, a key protein implicated in F-actin nucleation in filopodia (Witke, 2004).

The ablation of *Rac1* in MGE-derived IN progenitors at E13.5 induces a reduction of cIN progenitor proliferation due to defects in actin cytoskeleton organization, which prolongs the cell cycles, resulting in a 50% reduction of cINs in the postnatal cortex. Proliferating MGE-derived IN progenitors aggregate in their place of birth and fail to properly exit the cell cycle (Vidaki et al., 2012). Notably, *Rac1* is required for the transition from the G1 phase to S phase in MGE-derived progenitors as it regulates cyclin D protein expression and the phosphorylation of the retinoblastoma protein (Vidaki et al., 2012). The deletion of *Rac1* in postmitotic MGE-derived INs does not impact the final number of cINs in the mature cortex, suggesting that *Rac1* acts at the proliferation stage, but not during migration (Vidaki et al., 2012).

Rac3 (Ras-related C3 botulinum toxin substrate 3), another member of the Rac family, is highly expressed in the developing nervous system (Katayama et al., 2013). *Rac1* and *Rac3* double mutants display a significant loss of parvalbumin-expressing INs in the cortex and hippocampus, leading to reduced spontaneous inhibitory currents (IPSCs) and epilepsy (Tivodar et al., 2014; Vaghi et al., 2014). In *Rac1/Rac3* double-mutant mice, the usual migratory streams fail to form adequately and cINs travel shorter distances, while cell cycle exit is also delayed, resulting in a mixed proliferation and migration phenotype, ultimately decreasing the density of cINs in the mature cortex (Tivodar et al., 2014). In addition, a morphological defect is also observed, in part due to a decrease in acetylated tubulin, a post-translational modification involved in the stabilization of MT, resulting in a shorter but more branched leading process, together with impaired lamellipodia formation and reduced growth cone development (Tivodar et al., 2014). These morphological phenotypes likely contribute to the slowing of tangential migration. In addition, the dynamics of tangential migration is grossly impaired in dual *Rac1*/*Rac3* mutants, with decreased velocity, frequency and amplitude of translocations, as well as duration of migratory cycles and of leading process swelling (Kounoupa et al., 2023). The centrosome and Golgi complex are closer together and to the cell body in *Rac1*/*Rac3* mutants, correlating with shorter translocations (Kounoupa et al., 2023). Actomyosin contractility is also impaired, given reduced phosphorylation of MLC (pMLC) and decreased RhoA levels, leading to slower nuclear translocation in dual *Rac1*/*Rac3* mutants cINs (Kounoupa et al., 2023). This is partly due to MT instability as described before with the deletion of *Dcx* or nocodazole treatment impacting nuclear translocation (Tanaka et al., 2004; Baudoin et al., 2007). Finally, axonal outgrowth is defective in dual *Rac1*/*Rac3* mutants (Kounoupa et al., 2023), a process dependent on the activation of Rac1 by the EB1 (end binding1)-induced TRIO-NAV1 (neuron navigator 1) complex at the end of growing MT (van Haren et al., 2014). Notably, RNA sequencing in Rac1/Rac3 double mutant INs showed reduced expression of the two pore segment channel 2 (*Tpcn2*), a voltage-gated ion channel that mediates calcium release from lysosome-related stores upon activation by nicotinic acid adenine dinucleotide phosphate (NAADP) and c-jun N-terminal kinase (JNK; Calcraft et al., 2009; Jha et al., 2014; Lee et al., 2016; Ogunbayo et al., 2018). TPC2 is implicated in metastatic cell migration (Nguyen et al., 2017; Kounoupa et al., 2023). The pharmacological inhibition of *Tpcn2* by NAADP antagonists (trans-Ned19) in MGE-derived cINs induces a reduction in axon length and surface, as well as reduced nuclear translocation frequency, impairing migration (Kounoupa et al., 2023). Altogether, *Rac1* and *Rac3,* together with *Tpnc2* play key synergistic roles in cIN development by regulating their cell cycle progression as well as their migration and morphology.

The p21-activated serine/threonine kinase (PAKs) family, that includes 6 members (Pak1-6), are downstream effectors of Cdc42 and Rac1 (Hofmann et al., 2004). During neuronal migration, PAK1 activation induces the phosphorylation of LIM Kinase (LMK), which phosphorylates cofilin and tubulin cofactor B, both required for the reorganization of actin filaments and MT polymerization (Arber et al., 1998; Vadlamudi et al., 2005; Chen et al., 2011). PAK also phosphorylates myosin II by inhibiting MLC kinase (MLCK; Sanders et al., 1999). Mutations in PAK1 are associated with developmental delay, macrocephaly, and seizures (Cartwright et al., 2017; Hertecant et al., 2017; Harms et al., 2018; Horn et al., 2019). In cINs, *Dlx1/2* represses PAK3 to promote cell migration, while it is activated once cINs reach their final position when it regulates dendritic growth and postsynaptic differentiation (Cobos et al., 2007; Dai et al., 2014).

JNK (c-Jun N-terminal kinase) also acts downstream of Rac1/Pak1 signaling pathway (Kawauchi et al., 2003). JNK belongs to the mitogen-activated protein kinase (MAPK) superfamily, known to regulate several important physiological processes including cortical development and neuronal migration (Davis, 2000; Zhang et al., 2016a). JNK is regulated by the thousand and one amino-acid kinase 2 (TAOK2), involved in 16p11.2 duplication syndrome and schizophrenia (Davis, 2000; Coffey, 2014; Richter et al., 2019). The deregulation of the TAOK-JNK pathway impacts cINs by accelerating their development, as reported in 16p11.2 duplication mouse models (Willis et al., 2021). Notably, parvalbumin is downregulated in this model, an adaptative mechanism that aims to re-establish a proper balance between excitation and inhibition (Willis et al., 2021). The conditional deletion of *Jnk 1* in *Jnk 2* knockout mice impairs the morphology and tangential migration of cINs, with blurred migratory streams (MZ and SVZ/IZ), resulting in misplaced cINs (Myers et al., 2014). This occurs due to an early switch from tangential to radial migration and premature entry in the cortical plate (Myers et al., 2020). Further, the loss of *Jnk* in cINs leads to alterations of leading process branching dynamics through reduced growth cone splitting and shortening of the swelling extension in the leading process, together with an aberrant localization of the centrosome and primary cilium to the trailing process (Smith et al., 2020b). Overall, JNK plays critical roles in regulating cIN migration, downstream of Rac1/Pak1.

By contrast, RhoA is typically inhibited in migratory cells, including in cINs (Pacary et al., 2011). In this context, the deletion of *RhoA* in migrating cINs, after they exit the VZ, does not significantly impair their migration (Katayama et al., 2013). However, RhoA activation seems required during neurogenesis as its deletion in cINs progenitors in the VZ results in significant reduction of final cINs numbers (Katayama et al., 2013). Similarly, the deletion of Cdc42 in MGE-derived cINs does not alter their migration but it is required in the VZ for their normal differentiation (Katayama et al., 2013). Nonetheless, more studies are needed to understand the role of RhoA and Cdc42 in cIN migration since local activation of RhoA may be required to regulate actomyosin contractility and actin polymerisation that promote neurite and membrane retraction during migration (Etienne-Manneville and Hall, 2002; Ito et al., 2014). Notably, mDia, a downstream effector of RhoA, is actively involved in the nucleation and polymerization of actin (Higashida et al., 2004). Thus, deletion of *mDia1* and *mDia3* in cIN neuroblasts in mice results in striking impairments of tangential migration, with reduced distance between the centrosome and the cell body and decreased movements of the swelling in the leading process before nuclear translocations. On the other hand, *mDia* deficiency does not impair the radial migration of excitatory neurons (Shinohara et al., 2012), suggesting it plays a more selective role in tangentially migrating cINs.

Upstream regulators of RhoGTPases also play a critical role in neuronal development, including cIN migration. TRIO (Trio Rho Guanine Nucleotide Exchange Factor) is a dual GEF protein known to activate Rac1 and RhoA (Bellanger et al., 1998; Chhatriwala et al., 2007). *TRIO* mutations have been identified in patients with autism spectrum disorder, microcephaly, and intellectual disability, with or without epilepsy (Michaud et al., 2014; Sadybekov et al., 2017). While most described mutations are loss-of-function variants that selectively impact the GEFD1 domain, thus preventing Rac1 activation, some variants appear to induce a gain-of-function and enhanced Rac1 activation (Pengelly et al., 2016), and selected few variants involve the GEFD2-RhoA activating domain (Barbosa et al., 2020; Bonnet et al., 2023). Studies demonstrated that TRIO is critical for multiple aspects of brain development. In mice, complete knock-out of the gene is embryonically lethal while surviving mice show an aberrant cellular organization of the hippocampus and the olfactory bulb (O'Brien et al., 2000). Further, the deletion of *Trio* specifically in the nervous system induces a reduced brain size (microcephaly), with abnormal brain and hippocampal morphology, defective cerebellar granule cell migration and spatial learning deficits (Peng et al., 2010; Zong et al., 2015). At the cellular level, TRIO is known to regulate cell migration, axonal guidance, and dendritic development of pyramidal cells, mostly through its function as a Rac1 activator (Schmidt and Debant, 2014; Ba et al., 2016; Wei et al., 2022). However, recent evidence suggests that TRIO also plays critical roles in cIN migration and that targeted deletion in post-mitotic cINs suffices to induce autism-like behavior and epilepsy (Sun et al., 2021). Recent data indicate that the conditional deletion of *Trio* specifically in cINs alters the migration dynamics and morphogenesis of tangentially migrating cINs, with increased neurite complexity and reduced responses to guidance cues, resulting in impaired cortical inhibition and autism-like behaviors in mice (Sun et al., 2021).

DOCK7 (dedicator of cytokinesis 7), a member of the DOCK180 family, is another Rac GEF implicated in the differentiation and genesis of both pyramidal cells and cINs (Yang et al., 2012). Mutations in *DOCK7* are associated with epileptic encephalopathy and intellectual disability (Perrault et al., 2014). Recently, the loss of DOCK7 has been shown to disrupt the movement of the centrosome leading to slower tangential migration of the olfactory bulb INs (Nakamuta et al., 2017).

ARHGAP15 (GTPase-activating protein 15) is a GAP protein known to downregulate Rac1 and switch off the downstream signaling pathway (Seoh et al., 2003; Radu et al., 2013). Overexpression of this protein results in cell retraction due to the increase of stress fiber formation (Costa et al., 2011). *De novo* mutations in *ARHGAP15* have been associated with autism spectrum disorders and intellectual disability (O'Roak et al., 2011; Mulatinho et al., 2012). The targeted deletion of *Arhgap15* in cINs in conditional knock-out mice increases their susceptibility to seizures after treatment with pro-epileptic drug pilocarpine (Liaci et al., 2022). Furthermore, this deletion disrupts cIN migration, morphology and laminar distribution (Liaci et al., 2022). This suggests a critical role of these various GAPs in the development of inhibition by negative regulation of Rac1.

## 5. Clinical prospect of MGE-cIN progenitor transplants as cell-based therapies for neurodevelopmental disorders and epilepsy

Given the growing evidence that various monogenic forms of neurodevelopmental disorders, such as autism spectrum disorders, childhood epilepsy, severe developmental epileptic encephalopathies, intellectual disabilities and schizophrenia, may reflect primary disorders of cIN development, migration or function, the development of cell-based therapies involving the transplantation of cIN progenitors has gained momentum. Indeed, current pharmaceutical approaches are mostly symptomatic and have limited benefits, emphasizing the importance of exploring new therapeutic avenues. Thus, cell-based therapies, for instance transplantation of MGE-derived progenitors, have been extensively studied in animal models of genetic interneuronopathies, autism spectrum disorders and epilepsy (Anderson and Baraban, 2012; Hunt et al., 2013; Li et al., 2022; Righes Marafiga and Baraban, 2023). MGE-derived progenitors transplanted in neonatal and juvenile mice brains maintain an ability to migrate, disperse and integrate in the host circuits, often spanning great distances from the injection site, an interesting property when targeting multifocal complex circuits disorders (Alvarez-Dolado et al., 2006; Tong et al., 2014; Upadhya et al., 2019). Upon integration in the cortical circuitry, transplanted MGE-derived cINs selectively enhance local inhibition in a functionally relevant fashion (Wichterle et al., 1999; Alvarez-Dolado et al., 2006). Transplanted MGE-derived progenitors survive up to 1 year after transplantation, even in unfavorable environments (Zipancic et al., 2010; Tong et al., 2014; Lu et al., 2020). MGE-cell transplants have been shown to rescue behavioural deficits and prevent or reduce seizures in multiple mice models of autism-spectrum disorders or epilepsy (Alvarez-Dolado et al., 2006; Hunt et al., 2013). Additionally, MGE transplants are considered relatively safe, having minimal proliferative potential, compared to induced pluripotent stem cells (PSCs), which are prone to result in tumor formation (De la Cruz et al., 2011). Although ethical issues preclude the use of human-derived MGE cells, human-induced PSCs (hiPSCs) derived “MGE” cells are actively being considered, as are other mammalian sources of MGE cells (Righes Marafiga and Baraban, 2023).

## 6. Conclusion

Decades after the initial discovery of the origin of cortical interneurons in the subpallium of rodents, remarkable research efforts have helped advance our understanding of interneuron migration, focusing on identifying environmental guidance molecules as well as intrinsic factors implicated in this process. Yet, many questions remain open, awaiting further investigation. In particular, further studies are needed to clarify the intracellular signaling pathways activated by guidance cues in migrating interneurons as well as their impact on cytoskeletal remodeling. In addition, although some mechanisms of radial migration are shared between cINs and pyramidal neurons, such as gap-junction mediated attachment to an intact radial glia scaffold (Poluch and Juliano, 2007; Yokota et al., 2007; Elias et al., 2010), other mechanisms are specific for cIN radial migration, including attachment to vessels (Léger et al., 2020), inputs from thalamocortical projections (Zechel et al., 2016) and pyramidal cells (as detailed above). These cell-type specific molecular mechanisms guiding cIN radial migration must be further clarified. Further, the mechanisms underlying CGE- and POA-derived IN migration remain less well studied and must be further explored.

Most of our current knowledge about IN development and migration arises from animal studies using rodent models given limitations using human tissue. Although many aspects of cIN development, transcriptomic identity and migration are maintained across species (Ma et al., 2013; Krienen et al., 2020), some differences have been highlighted and it remains unclear to what extend evolution has altered these processes. For instance, the human brain has a much longer and complex developmental period compared to rodents. Primate studies found that cINs originate from both the ventral and dorsal forebrain, while only ventrally-derived cINs have been described in rodents (Jakovcevski et al., 2011; Hansen et al., 2013; Ma et al., 2013). How do these primate-specific cINs develop and migrate? What are the mechanisms involved? The development of hiPSCs and more recently forebrain assembloids (Birey et al., 2022) has opened new research avenues that will enable us to answer these questions as well as to further study human pathologies involving cIN development, so-called interneuronopathies, both from a mechanistic and translational point of view, using patient-derived INs. Nonetheless, testing these novel mechanisms and future therapies in whole animal models remains essential to provide adequate pre-clinical data for future therapeutic trials.

## Author contributions

IT: Conceptualization, Writing – original draft. AT: Conceptualization, Writing – original draft. ÉC: Writing – original draft. ER: Conceptualization, Funding acquisition, Resources, Writing – original draft.

## Funding

The author(s) declare financial support was received for the research, authorship, and/or publication of this article. This work was supported by the Canadian Institutes for Health Research (CIHR award #: PJT-173284). EC receives a Guy-Geoffroy Scholarship from the CHU Ste-Justine and PREMIER Scholarship from Montreal University. ER holds the Canadian Research Chair on the Neurobiology of Epilepsy and is a FRQS senior research scholar.

## Conflict of interest

The authors declare that the research was conducted in the absence of any commercial or financial relationships that could be construed as a potential conflict of interest.

## Publisher’s note

All claims expressed in this article are solely those of the authors and do not necessarily represent those of their affiliated organizations, or those of the publisher, the editors and the reviewers. Any product that may be evaluated in this article, or claim that may be made by its manufacturer, is not guaranteed or endorsed by the publisher.
